# Embodied Learning: Why at School the Mind Needs the Body

**DOI:** 10.3389/fpsyg.2019.02098

**Published:** 2019-10-01

**Authors:** Manuela Macedonia

**Affiliations:** ^1^Institute of Information Engineering, Johannes Kepler University Linz, Linz, Austria; ^2^Max Planck Institute for Human Cognitive and Brain Sciences, Leipzig, Germany; ^3^Linz Center of Mechatronics GmbH, Linz, Austria

**Keywords:** embodiment, instruction, education, second language, mathematics, memory, learning, neuroscience

## Abstract

Despite all methodological efforts made in the last three decades, Western instruction grounds on traditional principles. Most educational programs follow theories that are mentalistic, i.e., they separate the mind from the body. At school, learners sit, watch, listen, and write. The aim of this paper is to present embodied learning as an alternative to mentalistic education. Similarly, this paper wants to describe embodied learning from a neuroscientific perspective. After a brief historical overview, I will review studies highlighting the behavioral effectiveness of embodied instruction in second language learning, mathematics and spatial thinking. On this base, I will discuss some of the brain mechanisms driving embodied learning and describe its advantages, clearly pleading in favor of instructional practice that reunites body and mind.

## School Practice and Methods: Why It Is the Way It Is

School practice has been on the search for methods that should make instruction more effective for many decades (Reynolds et al., 2014). However, the scholar discussion on effectiveness has not reached practice in a satisfactory way. This is possibly due to the fact that the discussion is conducted on a pluralistic level (Scheerens, 2016). Also, the translation from scientific theory into classroom activities seems to be the major challenge and educators stay on what is called best practice (Bygate et al., 2013). They do their own teaching, i.e., a mix of what they have learned at university and what they have experienced during their professional development. This mix is shaped by restrictions due to time table, to compulsory learning materials like text books, and to other administrative factors that may differ from school to school type. They all have an impact on the outcome (Baker, 2014). At the end, despite all efforts, students still learn the way their parents have done, by reading and listening. This is the case for foreign languages (Stæhr, 2008). In maths, students learn to solve problems, as in the old days by learning formulas and applying them (Kilpatrick, 2014). In spatial thinking, i.e., the capacity to understand the relationship between space and objects in a dynamic way, at the base of technology and engineering, present instruction relies on capacities that students already have (Mahon and Hickok, 2016). For decades, psychometrics has been showing correlations between the ability to mentally visualize and manipulate spatial relationships and has attributed them to genetically determined factors. How to enhance these abilities on a large scale independently of genetic factors is left to improvisation, however (Uttal et al., 2012). Considering the proven connection between skilled spatial thinking, success at school and career choice, students with lower spatial abilities are disadvantaged in academic achievement. Furthermore, they are also not likely to embrace jobs in technology, considered the base of future innovation, as defined by the OECD as an economic and strategic goal for the future (OECD, 2012).

The reason why Western school instruction evolves slowly can be possibly searched in pedagogy. Pedagogy has deep roots into philosophy. Descartes (1596–1650) laid the foundations of the mind-body dichotomy in the Discours de La Méthode (Descartes, 1637). In his work, the French philosopher described on one side the body as a material “machine” containing organs and following the laws of nature. On the other side, Descartes described the mind as non-material and independent of the laws of nature. The interaction between body and mind would be enabled by the pineal gland, the “seat of the soul.”

The Cartesian dichotomy mind versus body has been put forth by philosophers as Locke and Kant, among others (Powell, 1990). More importantly, the influence of Dualism has continued in the 20th century because its principles have flown into Rationalism. There, the concept of “mind” is transposed into the concept of “reason,” i.e., the human capacity of thinking that derives from intrinsic intellectual structures of deduction (BonJour, 1997; Murphy, 2010). Rationalism claims that the only source of knowledge is the *a priori* reason. These thoughts are directly opposed to Empiricism. Empiricism grounds knowledge on the experience(s) that we collect with our senses, mediated by the body, *a posteriori* however.

Rationalism has strongly influenced cognitive sciences in the 20th century. Fodor et al. (1974) described the mind as a set of computational operations subdivided in modules that are defined in terms of their function. Originally, Fodor saw no connection between a module and the reference world outside. He separated the mind from the body in the manner of Cartesian philosophy. Later in his work, Fodor (1983) described the modules, language, for example, as separated from each other but interconnected with a central mechanism. Similarly, Pylyshyn (1984) described language as a phenomenon of the mind belonging to a system with amodal and symbolic units and deprive of connection to the reference objects outside. Last but not least, in Noam Chomsky’s most influential work, language was considered as an abstract phenomenon of the mind (Chomsky, 1965a, b, 1975). Again, also in Chomsky’s thought the body did not participate in cognitive processes.

Despite the fact that Fodor (2006, 2008), Pylyshyn (2007), Berwick et al. (2013), and Fodor and Pylyshyn (2014) have revised their positions, their original theories are still well eradicated in common sense. As such they had and have a great impact on learning and education. We “still” learn with the mind, the body contains our vital organs and allows us to move around. In other words, in our common sense, mind and body still subserve different functions. Hence, when acquiring knowledge, we sit quietly and concentrate on our “mental” task(s). In instruction, moving around while understanding or learning a subject is still considered as an experiment but not common practice.

## Embodied Cognition

With the advent of neuroscience, Rationalism has been greatly challenged. Theories of embodied cognition suggest that the mind is not an abstract and isolated entity. Rather the mind is integrated into the body’s sensorimotor systems (Barsalou, 1999, 2008). Followingly, cognitive processes are mediated by “body-based systems” (Alibali and Nathan, 2012), e.g., perception and action (Dijkstra and Post, 2015). Theories of embodied cognition hold that thinking of an object or a person triggers the simulation of the experience collected with the object or the person (Barsalou, 2008; Glenberg and Gallese, 2012). In fact, neuroscientific studies have demonstrated that processing of objects, spatial information, music, faces, flavors, odors, and also mere thinking of these concepts evokes sensorimotor responses, i.e., body-related activity in the brain (Pulvermuller, 1999, 2001; Pulvermüller, 2005). This relies on prior experience connected to manipulating objects, moving around, eating and smelling things (Zwaan, 1999). Areas and brain structures involved in the process wire together to networks of neurons (Hebb, 1949) that represent and store the information. For an apple that a person takes in her hand, neurons in visual and haptic areas connect to networks that represent shape, color and texture of the fruit (Buccino et al., 2016). Networks on different scales connect to other networks until the representation of the apple maps all experiences that the subject has collected related to the fruit. Thinking of an apple by activating the visual image, i.e., the shape of the fruit, will trigger other network components including the motor programs involved in grasping, lifting, peeling, smelling (González et al., 2006), tasting (Barrós-Loscertales et al., 2012), and chewing the fruit.

Cognitive neuroscience has validated behavioral evidence collected in the past. For example, linguistic meaning had been defined for decades in a syntactic way, i.e., as a relationship between phrase components. Kaschak and Glenberg and Kaschak (2002) came up with a novel view: phrase semantics has to do with the body. They asked subjects to identify a sentence containing a motion verb (“close the drawer”) as sensible and allowed them to perform either congruent or incongruent movements related to the phrase, i.e., an action away or toward the body, respectively. They found that participants performed better in the semantic task if the action was congruent. Glenberg and Kaschak proposed the action-compatibility effect (ACE) in order to explain why participants took advantage of congruent gestures.

The embodied view of cognition is grounded in sensory and motor experiences (Engel et al., 2013; Mahon and Hickok, 2016). They create multimodal sensorimotor representations in the brain (Barsalou, 2008). From a purely mechanistic view, cognition is thus the result of brain functions in a highly interconnected “system” of cells. These cells respond to stimulation, i.e., signals that come from the outside world via ears, eyes, skin, nose, tongue, and motor acts, hence from organs of the body. The “mind,” the “reason,” is performed by the brain, an organ of the body. Hence, the mind is not an abstract entity any longer.

## Embodied Language

One of the cognitive domains that have been most appealing to mentalistic theories is possibly language. In the seventies of the 20th century, Chomsky described it as an innate phenomenon of the mind expressed by symbols. Brain imaging studies in the last decades have shed a different light on language. For example, when participants lay quietly in the functional Magnetic Resonance Imaging (fMRI) scanner, simply reading action words like kick or pick, activates portions of motor cortices in the brain, specifically those controlling leg versus hand movements (Hauk et al., 2004). Similarly, reading odor words like jasmine or cinnamon elicits activity in olfactory regions of the brain (González et al., 2006). If words were “symbols,” abstract entities of the mind with no connection to the body, performing a mental task would not activate brain regions related to sensorimotor processes occurred during language acquisition and use.

In fact, if we observe how children acquire language, they perform a multitude of sensorimotor acts. Children hear and repeat sequences of sounds (words), i.e., symbols but these symbols are related to objects they perceive with their senses or to actions they perform. Children cannot be prevented from touching, dropping, smelling the objects and putting them in their mouths (Adams, 2016). Therefore, in the brain’s language, a word must be represented as a sensorimotor network that mirrors all experiences collected to the concept (Pulvermuller, 1999).

Strikingly, also abstract concepts have shown to be – at least to a certain extent – embodied. Borghi and Zarcone (2016) found that even abstract words evoke activity in the portion of the motor cortex related to the mouth. This is possibly due to linguistic-social information linked to the re-enactement of experience (Meteyard et al., 2012). In a recent paper, Buccino et al. (2019) explain that abstract words, if compared to concrete ones, are more difficult to brain image because their neural representation not only involves multiple biological effectors and different sensory systems but also brain areas coding for social context. This is to say that abstract words are also experience related but they are connected to highly complex experiential clusters. These clusters are not focally localized in a single region of the brain and therefore difficult to localize during stimulation.

Besides language (Glenberg and Gallese, 2012), sensorimotor networks in the brain represent also memory (Glenberg, 1997), perception (Leman, 2014), feelings (Niedenthal et al., 2009), and higher cognitive functions (Niedenthal and Barsalou, 2005). In other words, the mind is embodied in all of its parts to different degrees (Giummarra and Gibson, 2008; Pulvermüller, 2013). The problem of the grounding of symbols in the real world (Harnad, 1990) that could not be solved satisfactorily with mentalistic and symbolic approaches has found explanation in neuroscientific studies. All this is to say that mind and body are intertwined with each other and that Cartesian theories of the mind cannot be the reference for educationalists any longer.

## Second Language Learning (L2) and the Body

A first attempt to integrate the body in learning L2 was made in the Total Physical Response (TPR) developed by Asher in the 1960s (Asher, 1969, 1977). In the TPR, the teacher gave commands in L2. Learners listened, comprehended and executed the commands. The TPR did not require learners to speak at an early stage. This was done in order to take off pressure from language production. Learning L2 through commands was meant to simulate native language acquisition. In fact, children are asked by the caregivers to do things in order to interact with the adults’ world. The TPR did not focus on syntax and used mainly imperative verbs connected to the vocabulary. Consequently, verbs that could be put in the imperative form or communicative acts that do not need the imperative were not taught. Criticisms addressed the method as suitable for early stages of L2 acquisition in which vocabulary essentials can be acquired through action. Despite this, over the decades, the TPR made its way in practice because effective in supporting word recognition (Asher, 1969, 1977). However, no empirical investigations were conducted on the impact on memory for L2 words and phrases if learners perform commands while learning. The arguments in favor of the method have remained qualitative and descriptive in their nature.

Whereas in the TPR the connection L2 learning and the body was given via actions performed while listening to the trainer, other approaches use gestures to learn L2. Note that action and gesture are both tied to language but that they are not equal (Cartmill et al., 2012). Actions are movements of the body with an own goal. For example the action of locomotion from A to B can be labeled as “to go” or “to run” depending on the performance speed. Two fingers that mimic legs moving forwards do not have a locomotion goal *per se*. They do not perform an action to move from A to B. Rather, the moving fingers represent a concept that can be a verb as “to go, walk, run, stroll” but also a noun “walk, walker,” etc. In this case, the fingers perform gesture which is referred to thought (Goldin-Meadow, 2003). Gestures belong to different categories (McNeill, 1992) and have been used for centuries in L2 lessons. In his book, De Radonvilliers (1807) writes on methods to teach Latin to French natives. The author suggests the use of representative gestures to clarify concepts when association between L2 and first language is not possible and to avoid explanations in the native language (Larsen-Freeman and Anderson, 2013). In L2-literature, single or multiple gestures are described as pantomime (Carels, 1981; Seaver, 1992). They capture some aspects of concepts either iconically or metaphorically (Gärdenfors, 2017). Two hands opening an imaginary book can be the iconic gesture for “book” or for the verb “to read,” but also a gestural metaphor for an abstract concept as “theory” (Macedonia and Knösche, 2011). Emblems, like “thumb up” to express appreciation, are called conventional gestures. They are standardized and valid within a group of speakers. The meaning of an emblem can differ from culture to culture. For example the OK gesture is made by connecting the index finger and the thumb into a circle. In Anglo Saxon societies, this gesture denotes approval. Instead, in some Southern European countries, it is offensive and in the Arab world it is used while cursing (Kita, 2009). Finally, deictic gestures point to places (here, there, etc.) or to objects in the environment. Hence gestures, help us to communicate, they build with speech the two sides of the communicative coin, “they mutually interact to enhance language comprehension” (Kelly et al., 2010).

Interestingly, the explicative function of gestures is not new. Besides clarifying L2 word semantics, gestures fulfill a further purpose: they help to memorize vocabulary better than by only reading it or listening to it. This issue had already been described by Asher and Price (1967), for actions in a qualitative way, however. Quinn-Allen (1995) conducted the first empirical study on the impact of gestures on memory for words in L2. In a between-subjects study, she taught 112 English native students French expressions. One third of the group learned the expressions by reading them and saw no gestures at any time. The second third of the group memorized the expressions by reading them and performing emblematic gestures simultaneously. The third subgroup saw the gestures only in the test phase. The group that had learnt with emblematic gestures scored best in word recall. More interestingly, this group forgot fewer items than the group that had encoded without gestures. In her doctoral dissertation, Macedonia (2003) had participants learn thirty six items of an artificial corpus created for experimental purposes. Eighteen words were learned by hearing, reading and repeating the words aloud. For the other 18 words, representative gestures were first performed by the trainer live and thereafter imitated by the subjects. Significantly better results in word recall were obtained at all time points for those words that had been learned with the representative gestures. Similarly, in a study with the same learning conditions Macedonia and Knösche (2011) obtained comparable results. The authors used videos and audios instead of live performance and Vimmi, a severely controlled artificial corpus conforming with Italian phonotactics. Ninety-two items, composed in abstract sentences were learned audio-visually, half of which were additionally added metaphorical gestures in order to represent the words’ semantics. Words enriched by a gesture were better retained in the short but also in the long term. Another study by Kelly et al. (2009) on Japanese verbs that were learned audio-visually and by additionally performing a congruent or an incongruent iconic gesture yielded similar results. Better memorization was achieved with words trained with congruent gestures. Non-matching gestures did not empower memory. Along this line, further work comparing the recall of words learned with semantically related and semantically unrelated gestures showed that only semantically related gestures support memorization in the short and long term (Macedonia et al., 2011).

To this regard, three features have proven to be essential in order to get the memory results. First, gesture must be semantically related to the words. Second, learners must perform the gestures themselves. This has been described in the beginning of enactment research as the self-performed-task effect (SPT) (Engelkamp et al., 1994) and confirmed in various studies among which one with a pedagogical agent with anthropomorphic looks instead of a human (Macedonia et al., 2014a). In that study, school children aged of about 13 learned 45 Vimmi words. Fifteen items were encoded audio-visually, i.e., by reading the word and listening to an audio-file (AV), 15 were learned audio-visually and by observing the virtual trainer perform a semantically related gesture (AVO), and another 15 items were learned by additionally imitating the gesture performed by the avatar (AVOG). Compared to the baseline (AV) and to the AVO condition, the words were better retained if the gestures had been imitated, i.e., self-performed. Third, when learning with the body, the training must be massed. In fact, in studies that show the benefit of enactment on vocabulary items have trainings of a few hours a day, four or five days in which participants repeat the gestures actively (Macedonia and Knösche, 2011; Macedonia et al., 2011; Mayer et al., 2015, 2017). Other studies conducted with the same experimental materials and protocols showed reduced performance (Bergmann and Macedonia, 2013; Macedonia et al., 2014a) or performance that did not reach significance in the gestural condition (Macedonia et al., 2019). In those studies, participants were trained according to short experimental protocols with a few repetitions that occurred on one single day. Taken together, these studies indicate that the body is a powerful tool that helps the mind to acquire (at least) vocabulary in second language. Having in mind the considerations above and the research that has taken place in the last 20 years, it is obvious that words can no longer be understood as abstract symbols of the mind. Instead, neuroscientific research has proven that words are experience-related sensorimotor representations in our brains (Pulvermüller, 2005; Pulvermüller et al., 2005).

Why the body, why gestures support memory in the short and long term, is an issue that has not received enough attention yet. In 2001, Engelkamp advanced the hypothesis that motor actions can improve the storage of words because motor activity engages mechanisms of procedural memory in the learning process, additionally to declarative memory. In a combined behavioral and fMRI-study, Macedonia and Mueller (2016) let subjects hear and read words that they had learned previously learned with gestures. This stimulation was enough to activate multiple memory networks but most strikingly the motor cortices, the cerebellum, and the basal ganglia, i.e., structures involved in procedural memory circuits. In other words, the body not only represents knowledge but also is a powerful tool to additionally store knowledge. L2 instruction can definitely take advantage of techniques that involve procedural memory in order to enhance memory (Ullman and Lovelett, 2018).

## Mathematics and the Body

About two decades ago, cognitive science began to investigate the influence of co-speech gestures in mathematical concepts systematically, by observing gestures that are spontaneously produced by teachers. A seminal study by Susan Goldin-Meadow and co-workers on mathematical equivalence focused on the effect of spontaneous gestures accompanying speech in teachers and students (Goldin-Meadows et al., 1999). Teachers were instructed to provide solving strategies only orally, and by performing a gesture that was either matching (reinforced the message), or mismatching (differed from the verbal message). The gestures were embedded within the lesson. As it was hypothesized, children uptook significantly more often the strategy presented by the teacher if it had been accompanied by a matching gesture than by no gesture at all. Conversely, children did not benefit of the gestures conveying a solving strategy different from speech compared to speech only. Comprehension of the solving strategies was enhanced by matching and disrupted by mismatching gestures. Goldin-Meadows et al. (1999) related this results to a possible “second representational format” that gesture can provide. In other words, when speech and gestures are combined and match with each other, speech and therefore mathematical concepts are more easily captured because of the manual modality which is added to communication.

Similarly, Alibali and Nathan (2012) sustained that mathematical thinking is embodied. Following Mc Neills classification of gestures (McNeill, 1992), the authors analyzed the gestures produced by teachers and learners while explaining mathematical ideas and concepts. Thereafter pointing (deictic) gestures would ground thoughts in the physical environment, representational gestures would simulate perception and action, and metaphoric gestures would manifest conceptual metaphors.

In two experiments by Crollen and Noël (2015), 5 to 9-year-old children had to accomplish one-target, two-target counting tasks, and additions. While doing this, participants had no constraints, interfering hand movements, and interfering foot movements to deal with. Hand movements disrupted counting more than foot movements. These results suggest a connection between our fingers and counting. Considering that in childhood the acquisition of numerical skills is tightly connected with finger counting, these results do not surprise. In an fMRI study, Tschentscher et al. (2012) presented adults digits from 1 to 9 only visually while they lay quietly in the scanner. Dependently on the participants’ counting habits (left- or right-starters), hemodynamic activity in the contralateral motor cortex was observed when the numbers were presented. This neuroscientific evidence supports the view of embodied mathematical knowledge. The connection between motor activity in the brain and counting with fingers is connected to Hebbian learning mechanisms. They apply during learning and connect cognitive mathematical operations with finger movements performed while counting (Sato and Lalain, 2008).

Wagner (Cook et al., 2016) conducted a between-subjects study with 65 children (mean age 9 years) to investigate the effect of gesture learning on mathematical equation problems as “3 + 8 + 5 = 3 + 13.” The authors hypothesized that gestures would facilitate understanding of trained material and would promote transfer when children had to solve equations. In the study, an instructional avatar was employed in order to avoid confounding factors that can be created during live instruction delivered by a human. The avatar explained six equations either standing still or by performing additional gestures that were either representative (reinforcing the content) or beat gestures. The baseline condition consisted of a no-gesture condition, i.e., an explanation in which the avatar did not move at all. After instruction, children were assessed by means of equal addends equivalence problems with matching addends, transfer problems, and conceptual questions at the computer. In all tests, children who had learned from the gesturing avatar performed better than those children who had learned without gestures. Considering that this study was conducted with a computer animation avatar in a highly controlled environment, it stands to reason that the learning effect must come from the gestures themselves, as noted by the authors of the study in the discussion.

Peppler (2017) has recently described how counting is originated in the real world: We count sheep by observing them. If our eyes are shut or the sheep are not where we are, we retrieve a concrete image of the animals in order to count them. We don’t think of abstract living beings. Instead, we visualize sheep and not dogs the way we have experienced them. Furthermore, during counting, we use the body to support the cognitive task by counting on our fingers. We explain math basic operations (addition, subtraction, division, and multiplication) to children by putting real world things together, taking them apart from each other, cutting them, and so on. All this, we do by means of our body. Even alliterate persons who cannot read or write numbers can perform the operations by referencing the task to real world objects.

Nathan and Walkington (2017) have developed a theory of grounded and embodied mathematical cognition (GEMC) that proposes action and gesture as tools to understand properties of concepts related to science, technology, engineering, and mathematics (STEM). They investigated geometric proofs, an area in which conceptual understanding of abstraction is necessary and mathematical procedures (among others algorithms). According to Nathan et al. (2014), proof practice involves notations, speech, gesture, and enactment of mathematical concepts. In a behavioral study, 120 undergraduate students were asked to generate proofs by thinking aloud for two mathematical tasks in front of an interactive whiteboard. Before starting the proof, participants had performed either grounding (relevant to the task), or non-grounding (irrelevant) actions. For example, for a task concerning a triangle, the relevant action was to touch colored dots on the whiteboard that were symmetrically positioned in order to embody the key idea of triangle. Non-grounding actions like tapping on small diamonds on the whiteboard without any conceptual connection were also randomly performed. Grounding actions had a beneficial transfer effect on the experimental task: participants performed better in generating mathematical insights and could enhance their performance when additionally using language to describe the proof (Nathan et al., 2014).

## Implications for Instruction

There is a similarity in the way how we still consider both (second) language and mathematics: i.e., as abstract domains of cognition. Not only that we use the body in order to learn words and their meaning. We also learn mathematical concepts with the body and empirical evidence is cumulating year after year in favor of this view, of embodied mathematics (Soylu et al., 2018). In other words, the Cartesian era is over: neither Descartes, nor Kant or Fodor were right. Cognition is neither amodal nor symbolic. Empirical evidence shows that in at least two educational domains, i.e., second language and mathematics, embodied strategies lay the base for enhanced understanding and learning. The body - via action and gesture - is a powerful tool to understand and to learn school subjects. However, embodiment deprived of its neuroscientific base would have no chance in educational contexts. We have investigated the brain in order to know that the human mind does not work like a “computer” processing symbols. Neuroscience has unveiled (at least partially) brain patterns behind language and mathematical thinking and they are grounded in action and perception, in the body. The idea to pursue is now to create learning contexts which allow brain based instruction and embodied learning. These contexts can be natural, in interaction with a teacher but also they can employ immersive technologies, in which embodiment is performed in virtual or augmented reality (mixed reality) (Lindgren and Johnson-Glenberg, 2013; Macedonia et al., 2014b). Considering that the future of employment is tied to excellent education, there is an urgent need to make instructional methods more effective by combining evidence based behavioral and neuroscientific research with methodology.

## Author Contributions

The author confirms being the sole contributor of this work and has approved it for publication.

## Conflict of Interest Statement

The author declares that the research was conducted in the absence of any commercial or financial relationships that could be construed as a potential conflict of interest.
